# Modifier-Sensitive Phenotypic Divergence in XMEN Disease (MAGT1 Deficiency): Neurodegenerative and Immuno-Hematologic Trajectories

**DOI:** 10.3390/jcm15062395

**Published:** 2026-03-21

**Authors:** Ragip Fatih Kural, Zuleyha Galata, Reyhan Gumusburun, Ceyda Tunakan Dalgic, Nur Soyer, Havva Yazıcı, Ayse Nur Yuceyar, Aslı Subasıoglu, Irem Evcili, Bilgi Gungor, Kasım Okan, Mehmet Soylu, Cihat Uzunkopru, Omur Ardeniz

**Affiliations:** 1 Division of Allergy and Immunology, Department of Internal Medicine, Ege University Faculty of Medicine, Izmir 35100, Türkiye; zuleyhagalata61@gmail.com (Z.G.); reyhangumusburun@gmail.com (R.G.); drceydat@yahoo.com (C.T.D.); kasimokan55@gmail.com (K.O.); tanmehomu@yahoo.com (O.A.); 2 Division of Hematology, Department of Internal Medicine, Ege University Faculty of Medicine, Izmir 35100, Türkiye; drakadnur@yahoo.com; 3 Division of Pediatric Metabolism, Department of Pediatrics, Ege University Faculty of Medicine, Izmir 35100, Türkiye; havvaya@gmail.com; 4 Department of Neurology, Ege University Faculty of Medicine, Izmir 35100, Türkiye; nur.yuceyar@ege.edu.tr; 5 Department of Medical Genetics, Atatürk Training and Research Hospital, Izmir Katip Celebi University, Izmir 35360, Türkiye; asli.subasioglu@ikcu.edu.tr; 6 Izmir Biomedicine and Genome Center (IBG), Dokuz Eylül University, Izmir 35330, Türkiye; irem.evcili@ibg.edu.tr (I.E.); bilgi.gungor@ibg.edu.tr (B.G.); 7 Department of Microbiology, Ege University Faculty of Medicine, Izmir 35100, Türkiye; mehmet.soylu@ege.edu.tr; 8 Department of Neurology, Atatürk Training and Research Hospital, Izmir Katip Celebi University, Izmir 35360, Türkiye; cihat.uzunkopru@ikcu.edu.tr

**Keywords:** XMEN disease, MAGT1 deficiency, immunohematology, thrombotic microangiopathy, EBV-driven lymphoma, neurodegeneration, inborn errors of immunity, tailored surveillance

## Abstract

**Background:** X-linked immunodeficiency with magnesium defect, Epstein–Barr virus (EBV) infection, and neoplasia (XMEN) disease is a rare inborn error of immunity caused by loss-of-function mutations in *MAGT1*, leading to impaired N-linked glycosylation. Although chronic EBV viremia is a hallmark of XMEN disease, the mechanisms underlying its marked clinical heterogeneity remain poorly understood. **Methods:** We performed an in-depth clinical, immunological, and genetic characterization of two siblings carrying a pathogenic *MAGT1* variant (c.369_370insCC; p.Gly124fs), validated and deposited in ClinVar (SCV007293792). Assessments included whole-exome sequencing, multiparametric flow cytometry focusing on NKG2D expression, and longitudinal clinical follow-up. **Results:** Despite shared absence of NKG2D expression, the siblings exhibited strikingly divergent phenotypes. One sibling developed progressive neurodegeneration with central nervous system atrophy. The other presented with a complex immuno-hematologic phenotype, including EBV-positive Hodgkin lymphoma, recurrent autoimmune cytopenias, and lymphoma-associated thrombotic microangiopathy, representing a novel clinical association in XMEN disease. Comparative immunophenotyping revealed shared defects in B-cell maturation but distinct T-cell differentiation patterns. To contextualize neurological variability, we propose a descriptive, hypothesis-generating three-category conceptual classification comprising early-onset neurodevelopmental forms, adult-onset neurodegenerative manifestations, and secondary immune-mediated or vascular involvement of the nervous system. **Conclusions:** These findings demonstrate profound intrafamilial heterogeneity in XMEN disease and suggest a model in which modifier-sensitive factors influence organ-specific disease expression. The observation of lymphoma-associated thrombotic microangiopathy and the proposed descriptive neurological classification provide a conceptual framework that may help guide tailored, multidisciplinary surveillance beyond the primary genetic defect.

## 1. Introduction

X-linked immunodeficiency with magnesium defect, Epstein–Barr virus (EBV) infection, and neoplasia (XMEN) disease was first described by Li et al. in 2011 as a novel inborn error of immunity caused by loss-of-function mutations in the magnesium transporter 1 (*MAGT1*) gene [1]. MAGT1, located on the X chromosome, is essential for magnesium homeostasis in immune cells, and its deficiency results in combined immunodeficiency [1].

Earlier studies suggested that MAGT1 regulates T-cell activation through magnesium influx across the plasma membrane. This magnesium influx defect impairs CD8^+^ T-cell function and leads to chronic EBV viremia [1,2,3]. Later, MAGT1 was shown to localize to the endoplasmic reticulum, where it functions in N-linked glycosylation, a process critical for surface expression of immune receptors. Accordingly, MAGT1 deficiency is now primarily recognized as a congenital disorder of glycosylation [4,5,6].

MAGT1 functions as a non-catalytic subunit of the oligosaccharyltransferase (OST) complex in cooperation with STT3B (staurosporine- and temperature-sensitive 3B). Although it shares functions with its homolog, Tumor Suppressor Candidate 3 (TUSC3) [6,7], which is abundant in neural tissues and immune cells, the liver relies exclusively on MAGT1 for N-linked glycosylation. This tissue-specific distribution explains the phenotypic heterogeneity of XMEN and the predominant involvement of the immune system [5].

Loss of MAGT1 disrupts glycosylation, reducing surface expression of Natural Killer Group 2, member D (NKG2D) on CD8^+^ T cells and NK cells, as well as CD28 and CD70 on T cells and HLA-DRB on antigen-presenting cells. Reduced NKG2D expression is the hallmark of XMEN and represents the most reliable biomarker for diagnosis. These defects impair cytotoxic clearance of EBV-infected B cells, leading to chronic EBV viremia and a high risk of EBV-driven lymphoproliferation and lymphoma. Autoimmune cytopenias are also common. Beyond EBV, patients are susceptible to other viral infections, such as herpes simplex virus (HSV), varicella zoster virus (VZV), and molluscum contagiosum, as well as recurrent bacterial sinopulmonary and ear infections. Hypogammaglobulinemia or dysgammaglobulinemia with poor polysaccharide responses are frequent [4,8].

Beyond classic immune defects, XMEN also manifests with non-immune organ involvement, particularly in the liver, hematologic/coagulation systems, and the central nervous system (CNS) [8,9,10]. Despite the compensatory role of TUSC3 in neural tissue, neurological involvement was observed in approximately 30% of patients with MAGT1 deficiency (16/56) [9]. Neurological features have been reported, most notably intellectual and developmental delay, progressive neurodegeneration, as well as seizures and Guillain–Barré syndrome [6,11,12]. Reported neuroimaging findings include cerebral, cerebellar, brainstem, and spinal atrophy; basal ganglia and thalamic calcifications; cavum septum pellucidum; and multifocal white matter lesions [8]. Only a limited number of patients with XMEN have been reported to exhibit overt CNS atrophy [8,13,14]. The mechanisms underlying neurological involvement in MAGT1 deficiency remain poorly understood, and the factors contributing to its phenotypic variability remain unclear.

In this study, we performed an in-depth clinical, immunological, and genetic characterization of two male siblings who carried the same hemizygous *MAGT1* variant and exhibited markedly divergent disease trajectories. While one sibling developed progressive neurodegeneration with CNS atrophy, the other exhibited a complex immuno-hematologic phenotype characterized by EBV-driven Hodgkin lymphoma, recurrent autoimmune cytopenias, and the first reported instance of lymphoma-associated thrombotic microangiopathy (TMA) in XMEN disease. To contextualize these observations within a broader clinical framework, we propose a descriptive, hypothesis-generating three-tier conceptual classification of neurological involvement in XMEN disease, comprising Type I (neurodevelopmental), Type II (neurodegenerative and neuropsychiatric), and Type III (secondary central and/or peripheral nervous system involvement). This conceptual classification was informed by a narrative synthesis of previously reported cases and was applied to reorganize heterogeneous neurological manifestations into clinically meaningful categories. By integrating detailed immunophenotyping with clinical outcomes within this framework, this study aims to expand the phenotypic spectrum of XMEN disease and to provide insights into modifier-dependent organ-specific disease expression that may help structure personalized surveillance and therapeutic strategies.

## 2. Materials and Methods

### 2.1. Study Design and Ethical Considerations

This study was designed as an in-depth clinical and immunological characterization of two siblings diagnosed with XMEN disease, supplemented by a narrative literature synthesis, aiming to describe the intrafamilial phenotypic heterogeneity associated with a shared *MAGT1* genotype. This study was conducted in accordance with the Declaration of Helsinki. Ethical review and approval were waived for this study by the Institutional Review Board of Ege University (Exemption/Reference No: 2026-0552), as it constitutes a retrospective observational analysis of routine clinical diagnostic data. All genetic, immunological, and imaging analyses were performed as part of routine clinical diagnostic evaluation, and no additional research-specific interventions were conducted. Written informed consent for participation and publication was obtained from all patients.

### 2.2. Whole-Exome Sequencing and Variant Validation 

Genomic DNA was extracted from peripheral blood samples. Whole-exome sequencing (WES) was performed using Twist Biosciences technology. Briefly, approximately 36.5 Mb of Consensus Coding Sequences (targeting > 98% of RefSeq and Gencode v28 regions) were enriched from fragmented genomic DNA using the Twist Exome 2.0 kit (Twist Bioscience, South San Francisco, CA, USA). The resulting library was sequenced on an MGI DNBSEQ-G400 NGS platform (MGI Tech Co., Ltd., Shenzhen, China) to achieve a minimum read depth of 20× for >98% of the targeted bases, followed by alignment to the GRCh37/hg19 reference genome and variant calling using a validated bioinformatics pipeline. Variants were filtered based on population frequency and predicted functional impact, and interpreted according to the American College of Medical Genetics and Genomics (ACMG) guidelines [15]. The identified *MAGT1* and *COL4A1* variants were independently validated by Sanger sequencing using custom-designed primers and standard capillary electrophoresis. To definitively establish the de novo status of the *COL4A1* variant and assess segregation, targeted Sanger sequencing was performed on genomic DNA extracted from the proband’s peripheral blood and from both biological parents.

### 2.3. In Silico Variant Analysis

In silico analyses were performed to characterize the identified variants. Population allele frequencies were assessed using the Genome Aggregation Database (gnomAD) and dbSNP. Predicted deleteriousness of the *COL4A1* missense variant was evaluated using the Combined Annotation Dependent Depletion (CADD, v1.6) score, with Phred-scaled scores reported [16]. ClinVar and VarSome databases were queried to review prior submissions and reported clinical classifications. Evolutionary conservation of the affected amino acid residue was assessed using phyloP scores.

### 2.4. PBMC Isolation and Comprehensive Flow Cytometric Immunophenotyping

Peripheral blood samples were collected into EDTA-containing tubes from patients; age- and sex-matched healthy controls were included for the targeted NKG2D analysis only. Peripheral blood mononuclear cells (PBMCs) were isolated by density gradient centrifugation using Lymphocyte Separation Medium (LSM-A; Capricorn Scientific, Ebsdorfergrund, Germany) according to the manufacturer’s instructions. Briefly, whole blood was diluted 1:1 with sterile phosphate-buffered saline (PBS), carefully layered over the separation medium, and centrifuged without a brake (800× *g*, 20 min, room temperature). The PBMC layer at the plasma–LSM interface was harvested, washed twice, resuspended, and counted prior to downstream applications.

#### 2.4.1. Comprehensive Lymphocyte Subpopulation Analysis

Broad immunophenotypic profiling of lymphocyte subsets was conducted on isolated PBMCs. Surface and intracellular staining (utilizing PermWash buffer [eBioscience, San Diego, CA, USA] for permeabilization) were performed using appropriate fluorochrome-conjugated monoclonal antibodies. The extensive gating strategy encompassed total T, helper T, cytotoxic T, B, and NK cell populations. Detailed characterization of the B-cell compartment delineated naive, switched memory, IgM memory, transitional, and CD21^low^B-cell subsets, alongside plasmablasts. Furthermore, T-cell maturational profiling identified naive, central memory, effector memory, and terminally differentiated effector memory subsets within the CD4^+^ and CD8^+^ compartments, as well as follicular helper (Tfh) and γδ T cells (distinguished via TCRα/β and TCRγ/δ expression). Data for these comprehensive diagnostic panels were acquired using a Navios EX flow cytometer (Beckman Coulter, Brea, CA, USA) and analyzed via Kaluza software version 2.1. To ensure robust interpretation, reference intervals for these subsets were strictly adapted from established normative adult data: Besci Ö et al. [17] for general T and B subsets, and Zhang Y et al. [18] for Tfh cells. Routine complete blood count and biochemical parameters were evaluated against standardized institutional laboratory reference ranges.

#### 2.4.2. Targeted NKG2D Surface Evaluation

For the specific quantification of NKG2D expression, 1 × 10^6^ freshly isolated PBMCs were incubated with Zombie Aqua Fixable Viability Dye (BioLegend, San Diego, CA, USA) for 15 min at 4 °C in the dark to rigorously exclude non-viable cells. Following a wash step with FACS buffer (PBS containing 1% bovine serum albumin), cells were stained for 30 min at 4 °C in the dark with a dedicated fluorochrome panel: AF700 anti-human CD3 (clone SP34-2; BD Biosciences, San Jose, CA, USA), APC/Cy7 anti-human CD4 (clone OKT4; BioLegend), PE/Cy5 anti-human CD8 (clone HIT8a; BioLegend), APC anti-human CD16 (clone 3G8; BioLegend), FITC anti-human CD56 (clone HCD56; BioLegend), and PE anti-human NKG2D (clone 1D11; BioLegend). Cells were subsequently washed twice with FACS buffer and resuspended for acquisition. To ensure intra-assay reproducibility, all targeted NKG2D samples were acquired in duplicate. Data were collected using a NovoCyte flow cytometer (ACEA Biosciences, San Diego, CA, USA) and analyzed using NovoExpress software version 1.5.0.

#### 2.4.3. Quality Control and Quantitative Flow Cytometric Analysis

All flow cytometers were calibrated daily using manufacturer-provided quality control beads to ensure instrument stability. Fluorescence compensation was performed using single-stained controls. Gating strategies incorporated doublet exclusion, viability gating, and hierarchical lineage-based gating and were applied uniformly across patient and reference samples. Gating reproducibility was independently reviewed by two experienced operators.

For quantitative NKG2D assessment, both the percentage of NKG2D-positive cells and median fluorescence intensity (MFI) were recorded. MFI values were interpreted relative to age- and sex-matched laboratory reference samples processed under identical acquisition settings; relative fold reductions were calculated using the mean reference MFI as the technical baseline. The reference samples consisted of two age- and sex-matched adults without known immunological disorders and were included to provide a benchmark for signal interpretation. Duplicate acquisitions were performed for all targeted NKG2D analyses to assess intra-assay variability.

### 2.5. Humoral Immunity

Serum immunoglobulin levels and IgG subclasses were measured by nephelometry. Functional antibody responses were assessed by measuring specific IgG antibodies against pneumococcal polysaccharides using a commercial enzyme-linked immunosorbent assay (ELISA). According to the standardized reference ranges of the assay, antibody concentrations < 5 U/mL were defined as absent response, 5–7 U/mL as borderline, and >7 U/mL as positive (protective) response. Isohemagglutinin titers were determined using standard hemagglutination assays.

### 2.6. Virological and Laboratory Studies

EBV and cytomegalovirus (CMV) DNA loads were monitored longitudinally in whole blood (or plasma) using quantitative real-time PCR. Routine hematologic parameters were evaluated, and carbohydrate-deficient transferrin (CDT) testing was performed to assess systemic glycosylation abnormalities. Platelet function was assessed using standard platelet function analyzer assays.

### 2.7. Neuroimaging and Clinical Review

Brain magnetic resonance imaging (MRI) was performed on a 1.5-Tesla scanner (Magnetom Symphony, Siemens Healthineers, Erlangen, Germany) using standardized protocols including T1-weighted, T2-weighted, and fluid-attenuated inversion recovery (FLAIR) sequences. To provide a structured and semi-quantitative assessment of neurodegenerative changes, all neuroimaging data were retrospectively re-evaluated by senior neuroradiologists using the validated Global Cortical Atrophy (GCA) scale (Pasquier scale) [19].

The GCA scale is a visual rating system that assesses cortical sulcal widening and ventricular enlargement according to standardized criteria:

Grade 0: Normal cortical volume and no ventricular enlargement;
Grade 1: Mild sulcal widening and/or mild ventricular enlargement;
Grade 2: Moderate cortical volume loss and/or moderate ventricular enlargement;
Grade 3: Severe “knife-blade” cortical atrophy and/or severe ventricular enlargement.

Based on established normative data for age-matched cohorts, a GCA Grade of 0 is considered the healthy baseline for young adults, whereas a grade of ≥1 is defined as the pathological threshold for abnormal cortical atrophy in patients under 65 years of age [20]. A longitudinal semi-quantitative comparison was performed between the clinical MRI scans obtained in 2022 and 2024, enabling characterization of structural changes over a two-year interval.

### 2.8. Literature Analysis and Development of the Clinical Framework

To establish a structured approach to the neurological heterogeneity in XMEN disease, a narrative literature review was conducted in PubMed and Web of Science using predefined keywords related to “MAGT1,” “XMEN disease,” and “neurological involvement.” The search encompassed publications from 2011 (the first description of XMEN disease [1]) through December 2025. Titles and abstracts were screened to identify individual case reports and case series of genetically confirmed XMEN patients; approximately 60 published reports were reviewed descriptively. Among these, 18 individual patients with explicitly documented neurological manifestations (clinical and/or neuroimaging findings) were identified and included in the phenotypic synthesis. Including the index patient from our own cohort, a total of 19 patients were incorporated into the final conceptual framework.

Given the ultra-rare nature of XMEN disease and the marked heterogeneity and incompleteness of historical reports—frequently lacking detailed neurological examinations, dedicated neuroimaging protocols, standardized cognitive assessments, or longitudinal follow-up—a formal quantitative meta-analysis and standardized risk-of-bias assessment were not feasible. This constraint is consistent with the comprehensive systematic review by Golloshi et al. [9] and the descriptive case compilation by Benavides et al. [13], which identified neurological involvement in only 16/56 and 15/51 reported XMEN cases, respectively, underscoring the frequent absence of systematically collected neurological and radiological parameters across published reports.

Rather than attempting to aggregate isolated and inconsistently reported variables, the available clinical information was synthesized conceptually to identify recurring phenotypic patterns based on (i) age at onset, (ii) dominant neurological features (e.g., developmental delay, epilepsy, movement disorders, structural brain abnormalities), and (iii) overall disease trajectory. On this basis, cases were clustered into clinically meaningful categories to construct a pragmatic, hypothesis-generating three-tier classification framework (Type I–III), defined to maximize bedside discriminability and to align with practical surveillance implications. This framework is intended to support phenotypic stratification and to inform age-specific neurological surveillance and counseling in clinical practice, rather than to serve as a definitive, evidence-graded guideline. Accordingly, the surveillance suggestions are offered as pragmatic considerations rather than prescriptive recommendations.

### 2.9. Data Presentation and Statistical Approach

Given the ultra-rare nature of XMEN disease and the descriptive, intrafamilial comparative design, data are presented descriptively. No formal inferential statistical analyses were performed, as the primary objective was phenotypic characterization rather than inferential testing.

## 3. Results

### 3.1. Clinical Phenotypes and Intrafamilial Heterogeneity

**Figure 1:**
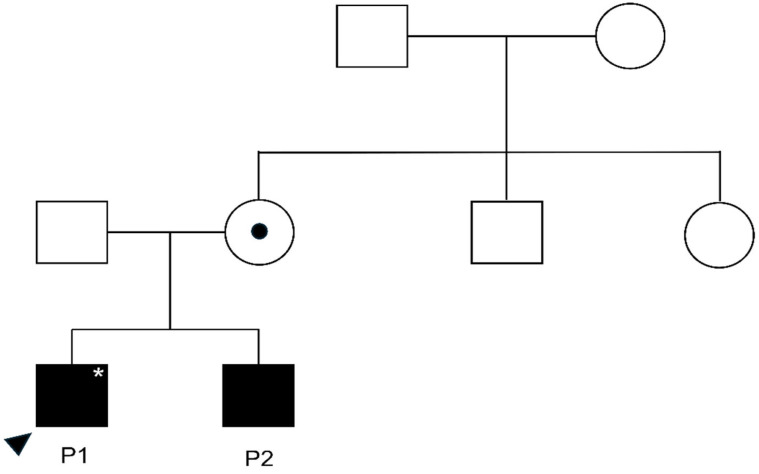
Pedigree of the family illustrating the segregation of *MAGT1* and *COL4A1* variants. Squares represent males and circles represent females. Filled symbols indicate individuals affected with XMEN disease carrying the hemizygous *MAGT1* frameshift variant (c.369_370insCC; p.Gly124fs), while the circle with a central dot denotes the heterozygous carrier mother. The proband (P1), indicated by an arrowhead and marked with an asterisk (*), additionally carries a heterozygous COL4A1 variant (c.3662C>T; p.Pro1221Leu), which is absent in the older sibling (P2) and both parents, confirming it as a de novo mutation.

The study cohort consisted of two male siblings from a non-consanguineous family, exhibiting markedly divergent clinical trajectories despite both carrying a hemizygous *MAGT1* frameshift variant (c.369_370insCC; p.Gly124fs). This pathogenic variant has been validated and deposited in ClinVar (Accession: SCV007293792). The family pedigree is shown in Figure 1, and Table 1 summarizes the intrafamilial phenotypic heterogeneity observed between the siblings. Segregation analysis confirmed the hemizygous *MAGT1* variant in both affected siblings and heterozygosity in the mother, whereas the maternal uncle tested negative; the maternal grandparents were not available for genetic testing. Furthermore, targeted Sanger sequencing of both parents confirmed the absence of the *COL4A1* variant, establishing its de novo status in the index patient (Patient 1).

### 3.2. Type II (Neurodegenerative) Phenotype: Patient 1

#### 3.2.1. Initial Presentation and Neuroimaging

**Figure 2:**
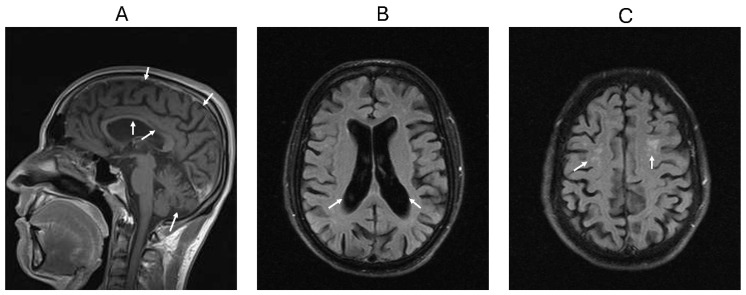
Brain magnetic resonance imaging demonstrating progressive cerebral and cerebellar atrophy in the neurologically affected sibling. (**A**) Sagittal T1-weighted image demonstrating diffuse cerebral and cerebellar atrophy with marked thinning of the corpus callosum (white arrows). (**B**) Axial T2-FLAIR image showing ventricular enlargement (white arrows). (**C**) Axial T2-FLAIR image revealing multifocal white matter lesions (white arrows).

The index patient (Patient 1) was previously healthy until the age of 27, when he developed unsteadiness and tremor while holding objects following a SARS-CoV-2 infection in 2022. Brain magnetic resonance imaging (MRI) demonstrated age-inappropriate cerebral and cerebellar atrophy, thinning of the corpus callosum, enlargement of the lateral ventricles, and multifocal white matter lesions (Figure 2), consistent with a Type II neurodegenerative phenotype. EEG was normal; CSF showed intrathecal IgG synthesis (IgG index 1.89) with CSF-restricted oligoclonal bands (Pattern II). Despite the temporal association with SARS-CoV-2, the absence of pleocytosis and normal CSF protein (33.7 mg/dL) made typical acute viral encephalitis unlikely. A broad serum paraneoplastic/autoimmune encephalitis antibody panel (Amphiphysin, CV2, Ma2/Ta, Ri, Yo, Hu, Recoverin, SOX1, Zic4, GAD65, Tr/DNER) was negative. Metabolic screening (quantitative tandem mass spectrometry amino-acid profiling), heavy-metal toxicology (lead, mercury, cadmium, arsenic), and CSF cultures were also unremarkable. Consequently, the isolated oligoclonal band positivity was considered more consistent with chronic intrathecal immune dysregulation in the context of MAGT1 deficiency, making common acute or post-infectious mimics of adult-onset neurodegeneration less likely.

#### 3.2.2. Genetic Findings 

Whole-exome sequencing revealed a hemizygous likely pathogenic variant in *MAGT1* (NM_001367916.1: c.369_370insCC; p.Gly124fs) and a variant of uncertain significance (VUS) in *COL4A1* (NM_001845.6: c.3662C>T; p.Pro1221Leu).

#### 3.2.3. Neurological and Radiological Progression

Approximately one year after the genetic diagnosis, neurological symptoms progressed despite physical therapy and rehabilitation, prompting referral to our institution for further immunological evaluation. Upon referral, neurological examination revealed dysarthria, lower limb weakness, dysmetria, ataxic gait, and impaired balance, indicating a progressive neurodegenerative course.

To further characterize structural evolution, the GCA/Pasquier scale was retrospectively applied to available longitudinal clinical MRI examinations. Both the 2022 and 2024 examinations were classified as GCA Grade 2 (moderate atrophy). Although the categorical grade remained unchanged, longitudinal visual comparison suggested progressive sulcal widening and ventricular enlargement over the two-year interval, consistent with within-category structural progression.

#### 3.2.4. Immunological Profile and Management

**Figure 3:**
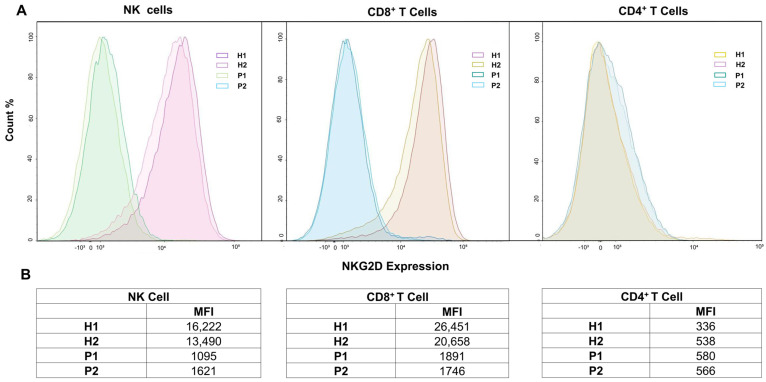
NKG2D expression on lymphocyte subsets in XMEN patients and healthy controls. (**A**) Representative overlaid flow cytometry histograms depicting NKG2D surface expression on NK cells, CD8^+^ T cells, and CD4^+^ T cells obtained from two healthy controls (H1, H2) and the affected siblings (P1, P2). Fluorescence intensity is shown on a logarithmic scale on the x-axis, while the y-axis represents normalized event frequency (Count %). (**B**) Corresponding quantitative assessment detailing the Median Fluorescence Intensity (MFI) values for each subject across the analyzed lymphocyte subsets. The tabulated data quantitatively substantiate the visually profound reduction in NKG2D expression on NK and CD8^+^ T cells in both patients compared to healthy controls, alongside the predictably low baseline expression on CD4^+^ T cells across all groups. Furthermore, relative fold-reduction calculations (using the mean MFI of healthy controls as a reference) demonstrated approximately 12- to 13-fold decreases in CD8+ T cells and 9- to 13-fold decreases in NK cells in the affected siblings.

Although the patient had no history of severe infections, he failed to respond to the pneumococcal polysaccharide vaccine. Serum immunoglobulin levels were normal, but B-cell immunophenotyping showed an expansion of naïve B cells and a reduction in memory B cells (Table 2). Crucially, flow cytometric analysis demonstrated absent NKG2D expression on both CD8^+^ T cells and NK cells (representing an approximate 9- to 13-fold quantitative reduction relative to healthy controls), confirming the diagnosis of XMEN disease (Figure 3). Additional investigations showed chronic EBV viremia and impaired platelet function, though no bleeding diathesis was observed. CDT was normal. Intravenous immunoglobulin (IVIG) replacement therapy was initiated due to the specific antibody defect.

### 3.3. Immuno-Hematologic Phenotype: Patient 2

#### 3.3.1. Initial Presentation and Malignancy

The 34-year-old male sibling of the index patient had immune thrombocytopenia (ITP) at the age of 7, which subsequently entered complete remission. He remained clinically stable until the age of 29, when he presented with jaundice, severe anemia, and thrombocytopenia. The detection of fragmented erythrocytes on peripheral smear, together with a negative Coombs test, raised the suspicion of TMA, and therapeutic plasma exchange was initiated. Crucially, laboratory evaluation showed normal renal function and preserved ADAMTS13 activity, thereby excluding classical hemolytic uremic syndrome (HUS) and thrombotic thrombocytopenic purpura.

During the same hospitalization, right axillary lymphadenopathy was identified, and excisional lymph node biopsy confirmed nodular lymphocyte-predominant Hodgkin lymphoma (NLPHL, stage 3A). Immunohistochemical analysis demonstrated that the Hodgkin/Reed–Sternberg cells were positive for EBV by EBV-encoded small RNAs (EBER) in situ hybridization. In the setting of concomitant EBV viremia and EBV-positive lymphoma, the clinical picture was compatible with lymphoma-associated TMA, representing a novel immuno-hematologic manifestation of XMEN disease. This patient had previously been reported as possible atypical HUS [21]; however, our longitudinal evaluation has reclassified this presentation. He was treated with six cycles of rituximab, cyclophosphamide, doxorubicin, vincristine, and prednisone (R-CHOP) without radiotherapy and achieved complete remission within one year, with resolution of cytopenias.

#### 3.3.2. Recurrent Immune Dysregulation

Following remission, the patient experienced a period of severe immune dysregulation starting at age 31. He developed pneumonia followed by Coombs-positive autoimmune hemolytic anemia (AIHA), which required multiple lines of therapy including corticosteroids, IVIG, and rituximab. Recurrent hemolytic episodes were often triggered by infections. Subsequently, at age 32, he developed another episode of ITP, completing a clinical picture consistent with secondary Evans syndrome. Serial monitoring revealed concurrent EBV and CMV viremias, necessitating antiviral therapy with ganciclovir for progressive CMV replication. He eventually attained complete remission and has remained disease-free for over two years.

#### 3.3.3. Genetic Confirmation and Immunological Profile

WES performed at the age of 34, following the diagnosis of XMEN disease in his sibling, identified the same hemizygous *MAGT1* variant. Immunological evaluation demonstrated mildly decreased serum IgG, IgG1, and IgM levels (Table 3). B-cell immunophenotyping revealed a pattern consistent with the index patient (expanded naïve B cells, reduced memory B cells). Meanwhile, T-cell analysis showed a low CD4/CD8 ratio and increased effector memory CD4^+^ T cells (Table 2), likely reflecting the burden of chronic viral surveillance and prior malignancy. Flow cytometry confirmed the absence of NKG2D expression, consistent with XMEN disease (Figure 3).

#### 3.3.4. Current Clinical Status

Unlike his sibling, neurological examination and brain MRI findings were unremarkable, with no evidence of neurodegeneration (GCA Grade = 0). However, imaging revealed chronic sinusitis and bronchiectasis. Platelet function assays were impaired, similar to the index patient, though no bleeding history was reported. Considering the burden of recurrent infections and immune dysregulation, tailored IVIG replacement therapy was initiated. The patient remains clinically stable under multidisciplinary immuno-hematological follow-up, with strict surveillance for infectious complications, cytopenias, and lymphoproliferative recurrence.

## 4. Discussion

XMEN disease is a combined immunodeficiency and a congenital disorder of glycosylation, characterized by multisystem involvement and marked phenotypic heterogeneity [4]. While chronic EBV viremia and immune dysregulation constitute its canonical features, neurological involvement has emerged as an underrecognized but clinically relevant dimension of the disease [8,9]. In this study, we describe two siblings who carry the same hemizygous *MAGT1* variant and exhibit persistent EBV viremia yet follow strikingly divergent clinical trajectories. One sibling developed progressive neurodegeneration with central nervous system atrophy, whereas the other presented with a complex immuno-hematologic phenotype dominated by immune dysregulation and EBV-associated hematologic malignancy. This intrafamilial comparison highlights the pronounced variability of organ-specific disease expression in XMEN and suggests that the shared *MAGT1* variant alone may not fully account for the divergent clinical trajectories observed within this family.

Progressive neurodegeneration with CNS atrophy has been described only rarely in XMEN [13,14]. The neurologically affected sibling demonstrated marked CNS atrophy together with a gradual neurological decline, placing him among the few patients with this severe phenotype. Crucially, this distinct neurodegenerative presentation served as the sentinel event that triggered genetic investigation, ultimately solving the diagnostic odyssey for his sibling (Patient 2), whose clinical course had evolved from childhood-onset ITP to severe, complex immuno-hematologic complications in adulthood. 

To contextualize the heterogeneity of neurological involvement reported to date, we propose a descriptive, conceptual three-tier clinical classification based on age at onset and dominant neurological features. Type I comprises childhood-onset neurodevelopmental involvement characterized by intellectual disability and developmental delay. Type II, which includes our index patient, represents an adult-onset neurodegenerative phenotype with progressive cerebral and/or cerebellar atrophy. Type III encompasses secondary involvement of the central and peripheral nervous systems due to immune-mediated, infectious, or vascular causes. These categories are not mutually exclusive, as a single patient may develop a combination of these types over their disease course. For instance, as observed in our index patient, the chronic progressive atrophy of Type II may present with an acute clinical deterioration, closely mimicking the acute secondary events of Type III. Despite these clinical complexities, this framework provides a descriptive approach to distinguish primary neurodevelopmental involvement from adult-onset neurodegeneration, which may help guide tailored anticipatory neurological surveillance, as outlined in Table 4. Consequently, we propose that the presence of these suggestive neurological patterns in patients with unexplained lymphoproliferation or cytopenias should serve as a clinical ‘red flag’ prompting genetic evaluation for XMEN disease.

The mechanisms underlying neurological involvement in XMEN disease remain incompletely understood. Functional overlap between MAGT1 and its homolog TUSC3 in neural tissue may provide partial compensation in most individuals, yet this compensation appears insufficient in a subset of patients, resulting in severe neurological disease. MAGT1 is an essential subunit of the STT3B complex and plays a critical role in post-translational N-linked glycosylation [6].

CDT is often utilized as an accessible, supportive biomarker for systemic N-glycosylation abnormalities in XMEN disease [8]. However, CDT has imperfect sensitivity [23,24] and may remain normal despite significant MAGT1-related glycosylation defects. Accordingly, the normal CDT values observed in our patients do not exclude a glycosylation abnormality; rather, they underscore that NKG2D surface expression, coupled with molecular confirmation, remains the most robust diagnostic readout for XMEN.

Given the prominent expression of MAGT1 in the vascular endothelium [25], we hypothesize that its deficiency may destabilize the neurovascular unit by impairing the glycosylation of key structural components. Specifically, this defect likely compromises both the folding of basement membrane proteins (e.g., type IV collagen, laminins) [26] and the biosynthesis of the endothelial glycocalyx [27]. This combined structural deterioration could establish a background of vascular vulnerability that facilitates neuroinflammation; however, further studies assessing endothelial integrity are required to substantiate this potential pathomechanism.

Notably, WES revealed a COL4A1 VUS exclusively in the neurologically affected sibling. Although classical COL4A1-associated phenotypes, such as porencephaly or major intracerebral hemorrhage, were absent [28], we explored the hypothetical biological relevance of this finding given the spatial co-expression of MAGT1 and COL4A1 in human brain tissue (Supplementary Figure S1). The variant is ultra-rare, evolutionarily conserved, confirmed de novo by parental testing, and exhibits a high CADD score, supporting predicted deleteriousness (Supplementary Table S1). In the context of MAGT1 deficiency–associated glycosylation defects, we propose a theoretical “synergistic hit” model in which impaired basement membrane integrity and reduced endothelial resilience might lower the threshold for a *COL4A1* variant to manifest clinically. Although its confirmed de novo status and supporting in silico predictions strengthen its potential biological relevance, without functional validation assays, this proposed interaction remains a speculative, rather than definitive, explanation for the divergent neurological outcomes between the siblings.

This concept of glycosylation-dependent vascular vulnerability extends beyond the central nervous system and may also contribute to systemic immuno-hematologic manifestations in XMEN disease. EBV-driven lymphomas represent the major source of morbidity in XMEN, affecting up to 50% of patients by the third decade. Classical Hodgkin lymphoma was the most commonly observed malignancy in XMEN [8]. In contrast, the hematologically affected sibling in our study developed EBER-positive nodular lymphocyte-predominant Hodgkin lymphoma (NLPHL), a notable finding given that NLPHL is typically an indolent and EBV-negative malignancy, with EBV positivity restricted to a very small subset of cases [29]. This unexpected EBV positivity should prompt suspicion of an underlying inborn error of immunity. Indeed, without his sibling’s sentinel neurological presentation, this patient’s MAGT1 deficiency might have remained undiagnosed. 

This patient initially presented with microangiopathic hemolysis and later developed AIHA and ITP consistent with a secondary Evans syndrome. This patient had been previously described in a letter to the editor as a possible case of atypical HUS in the context of EBV-positive Hodgkin lymphoma [21]. However, the sustained remission after chemotherapy and the more detailed immunological characterization in our study supported the diagnosis of lymphoma-associated TMA rather than aHUS. While TMA is a well-recognized complication of certain aggressive malignancies [30], to our knowledge, TMA has never been reported in association with NLPHL. The occurrence of severe microangiopathy as the presenting feature of a classically non-aggressive lymphoma is highly atypical. Rather than attributing the TMA solely to the lymphoma, we propose that the underlying MAGT1 deficiency provides a critical prothrombotic predisposition. Recent evidence demonstrates that MAGT1 deficiency profoundly disrupts hemostasis, leading to severe platelet dysfunction and a marked prothrombotic phenotype [31,32]. Therefore, to our knowledge, this represents the first reported association of lymphoma-associated TMA with XMEN disease. 

This unusual presentation reinforces the hypothesis of a shared background of endothelial vulnerability in XMEN disease, linking the vascular fragility observed in the CNS to systemic hematologic microangiopathy. However, we acknowledge that this mechanistic link remains speculative without functional endothelial validation. Consequently, this systemic endothelial vulnerability should be regarded as a theoretical, hypothesis-generating model.

Beyond organ-specific manifestations, the core immunophenotype remains a defining feature of XMEN disease. Systematic reviews have described a characteristic immune profile, including elevated B-cell counts, a reduced CD4/CD8 ratio, expansion of double-negative T cells, CD4 lymphopenia, and a predominance of naïve B cells with reduced class-switched memory subsets; hypogammaglobulinemia or dysgammaglobulinemia may also occur [8,9]. In our comparative analysis, B-cell subset percentages were similar between siblings, whereas T-cell phenotypes diverged markedly. The neurologically affected sibling retained a higher proportion of naïve T cells, whereas the hematologically affected sibling exhibited expanded effector memory populations, consistent with chronic immune activation driven by malignancy and viral replication. These findings illustrate how shared genetic defects may give rise to distinct immune trajectories aligned with the dominant organ phenotype.

**Figure 4:**
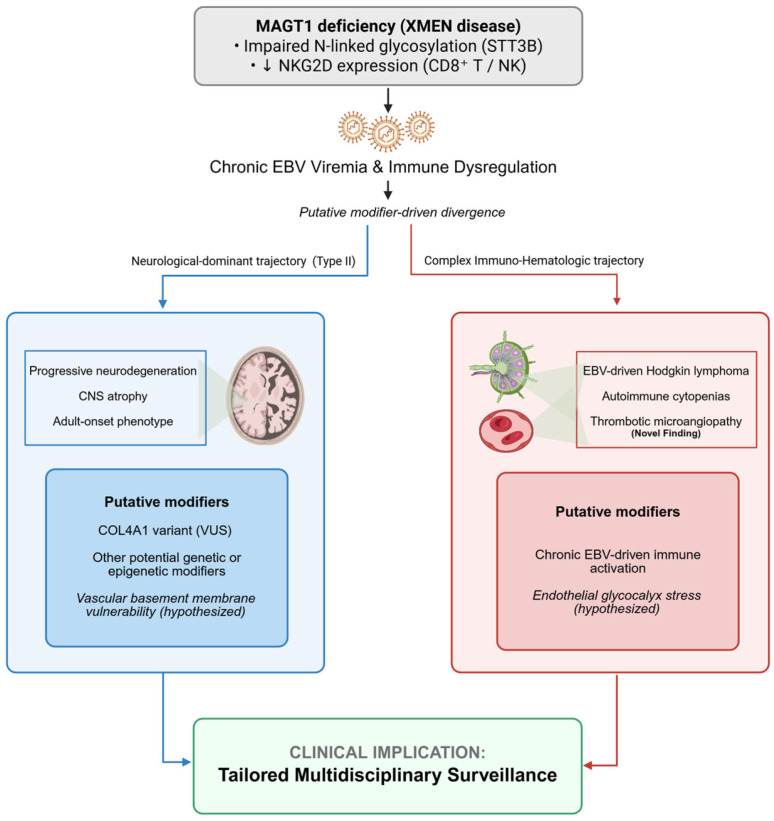
A proposed, hypothesis-generating model of modifier-driven phenotypic divergence in XMEN disease. The diagram summarizes a theoretical framework in which shared MAGT1 deficiency and chronic EBV viremia may be associated with distinct neurological and immuno-hematologic trajectories, potentially shaped by additional modifiers (e.g., a *COL4A1* variant). Note: Mechanistic links—particularly those related to vascular/endothelial susceptibility—are presented as biologically plausible hypotheses rather than empirically demonstrated defects in our cohort. The downward arrow (↓) indicates decreased expression. CNS, Central Nervous System; EBV, Epstein-Barr Virus; NK, Natural Killer; NKG2D, Natural Killer Group 2, member D; STT3B, Staurosporine-and-temperature-sensitive 3B; VUS, Variant of Uncertain Significance. Figure created with BioRender.com.

Taken together, these observations emphasize that XMEN disease unfolds along modifier-sensitive, organ-dominant trajectories rather than a uniform clinical course. Although a single trajectory may dominate at presentation, patients may carry latent risk for alternative organ involvement over time. Accordingly, long-term management should extend beyond infection control alone and incorporate vigilant, tailored multidisciplinary surveillance, including regular neurological assessment, EBV monitoring, and screening for autoimmune and lymphoproliferative complications. A visual summary of this proposed, hypothesis-generating model of modifier-sensitive, organ-dominant disease trajectories is provided in Figure 4.

Current management of XMEN remains largely supportive, with allogeneic hematopoietic stem cell transplantation representing the only established curative option, albeit associated with substantial treatment-related mortality and severe bleeding and thrombotic events [33]. MAGT1 deficiency impairs platelet function and N-linked glycosylation of key glycoproteins, thereby predisposing to hemorrhagic and thrombotic complications [31]. Emerging strategies such as epigenetic activation of TUSC3 [34] and CRISPR-based correction of MAGT1 [35] remain investigational and were not evaluated in the present study; they are cited here solely for context. These observations also motivate consideration of potential endothelial vulnerability in future studies, although this was not directly assessed in our cohort.

This study has limitations inherent to ultra-rare disorders. While intrafamilial comparison provides a controlled genetic background, the sample size is limited. Crucially, although targeted Sanger sequencing of both parents supports the de novo occurrence of the *COL4A1* variant, formal genetic confirmation of biological parentage was not performed, which remains a technical limitation. Furthermore, it remains classified as a VUS, which constitutes an important limitation because no functional validation is currently available. In addition, the absence of targeted functional assays (e.g., collagen secretion studies or in vivo variant-assessment models for this COL4A1 change), as well as the lack of specialized endothelial and microangiopathy biomarkers (such as soluble thrombomodulin or vWF multimers), currently precludes any definitive claims of causality. Our mechanistic models should therefore be regarded as strictly speculative and hypothesis-generating. Moreover, prior glycoproteomic studies have largely focused on lymphocytes, potentially underrepresenting defects in tissues with high secretory demand, such as the endothelium. Finally, neurological involvement may also have been underrecognized in earlier reports due to non-standardized assessments, and the inability to perform quantitative 3D MRI volumetrics with normative z-scores in our retrospective cohort further limits standardized structural comparison. Consequently, our proposed three-tier neurological classification remains a conceptual framework that necessitates independent validation before it can be considered an operational clinical tool.

## 5. Conclusions

This comparative analysis of two siblings with XMEN disease demonstrates striking intrafamilial divergence, with distinct neurodegenerative and complex immuno-hematologic trajectories arising from a shared genetic background. Beyond highlighting the role of modifier-dependent mechanisms, we describe lymphoma-associated TMA as a novel clinical association observed in the context of XMEN disease, supporting the hypothesis of potential systemic endothelial vulnerability. The proposed neurological classification provides a descriptive, hypothesis-generating conceptual framework for phenotypic characterization and may help structure anticipatory surveillance. Future studies should prioritize functional endothelial assessments to directly investigate the hypothesized vascular vulnerability and aim to externally validate the proposed neurological classification in larger, independent cohorts. Ultimately, early genetic diagnosis, vigilant longitudinal follow-up, and tailored multidisciplinary care are essential to optimize outcomes in this complex disorder.

## Figures and Tables

**Table 1:** Intrafamilial phenotypic heterogeneity in two siblings with XMEN disease.

**Neurological Findings**	**Patient 1**	**Patient 2**
Cognitive Decline	✓	–
Dysarthria	✓	–
Dysmetria	✓	–
Ataxia (gait and balance impairment)	✓	–
CNS atrophy	✓	–
Corpus callosum thinning	✓	–
Cerebral white matter lesions	✓	–
Cavum septum pellucidum	–	–
Intracranial calcifications	–	–
**Hematologic Findings**		
Lymphadenopathy	–	✓
Splenomegaly	–	✓
EBV-driven lymphoma	–	✓
Autoimmune Cytopenias (AIHA/ITP)	–	✓
Impaired platelet function assays	✓	✓
**Infectious susceptibility and complications**		
Recurrent Sinopulmonary Infections	–	✓
Chronic Sinusitis	–	✓
Bronchiectasis	–	✓
**Other organ involvement**		
Elevated Transaminase (ALT)	✓	–
**Treatment**		
IVIG replacement therapy	✓	✓

AIHA: Autoimmune Hemolytic Anemia; CNS: Central Nervous System; EBV: Epstein–Barr Virus; ITP: Immune Thrombocytopenia; IVIG: Intravenous Immunoglobulin. Note: (✓) indicates the presence of the clinical finding; (–) indicates its absence.

**Table 2:** Comparative immunological and flow cytometric findings in two siblings with XMEN disease.

Parameter	Patient 1	Patient 2	Normal Range
CD3^+^ (cells/µL)	1690	1153	998–5625
CD19^+^ (cells/µL)	**1212**	223	87–541
CD4/CD8	1.4	**1**	1.3–2.6
CD3^+^CD4^+^ (cells/µL)	893	**502**	673–3110
CD3^+^CD8^+^ (cells/µL)	606	502	238–1570
CD16^+^56^+^ (cells/µL)	255	465	91–766
TCRαβ^+^ CD4^−^ CD8^−^ (DNT), %	1.2	1.2	0.5–3.9%
TCRγδ^+^ T cells, %	7	**13.8**	0.6–12.3%
**Percentage of TCRαβ^+^ CD4^+^ T cells**			
CD4^+^CD45RA^+^CCR7^+^ (Naive), %	42.9	**6.1**	13.9–66.4%
CD4^+^CD45RA^−^CCR7^+^ (CM), %	27.1	48.4	21.7–67%
CD4^+^CD45RA^−^CCR7^−^ (EM), %	**29**	**44.9**	2.9–24.6%
CD4^+^CD45RA^+^CCR7^−^ (TEMRA), %	0.8	0.4	0.3–44.6%
CD4^+^CXCR5^+^ (Tfh), %	8.4	**10.2**	1.8–8.9%
**Percentage of TCRαβ^+^ CD8^+^ T cells**			
CD8^+^CD45RA^+^CCR7^+^ (Naive CD8^+^), %	46	23.4	4.1–67.5%
CD8^+^CD45RA^−^CCR7^+^ (CM), %	8.9	6.7	1.4–27.9%
CD8^+^CD45RA^−^CCR7^−^ (EM), %	16.9	**40.1**	0.7–39.9%
CD8^+^CD45RA^+^CCR7^−^ (TEMRA), %	7.9	29.7	3.6–66%
**Percentage of B cells**			
CD19^+^IgD^+^CD27^−^ (Naive B), %	**92.5**	**95.7**	33.7–79.2%
IgD^+^CD27^+^ (IgM memory), %	**1.9**	**1**	5.3–31.6%
CD19^+^IgD^−^CD27^+^ (Switched memory B), %	**3.2**	**1.4**	5.9–34.5%
CD21^low^ B Cells, %	**0.8**	**0.8**	1.2–14.2%
IgM^++^IgD^++^CD24^++^CD38^++^ (transitional B cells), %	0.6	3	0.6–3.5%
CD20^−^ CD38^++^ CD27^+^ (plasmablasts), %	0.7	0.4	0.4–3.6%

CM: Central Memory; DNT: Double-Negative T cells; EM: Effector Memory; NK: Natural Killer; TCR: T-Cell Receptor; TEMRA: Terminally Differentiated Effector Memory re-expressing CD45RA. Note: Bold values indicate results outside the reference range. Flow cytometry–based lymphocyte subpopulation reference ranges were adapted from age-matched normal values in the literature [17,18].

**Table 3:** Laboratory Findings in Two Patients with XMEN Disease.

Parameter	Patient 1	Patient 2	Normal Range
White blood cell count	6.32	7.23	4.5–11.0 × 10^9^/L
Absolute lymphocyte count	**3.78**	**3.83**	0.65–2.80 × 10^9^/L
Platelet count	175	171	150–450 × 10^9^/L
PFA Collagen/ADP	**218**	**141**	68–121 s
PFA Collagen/Epinephrine	**>260**	**>300**	84–160 s
ALT (U/L)	**74**	17	0–40
AST (U/L)	34	19	0–40
IgG (g/L)	8.19	**5.06**	7–16
IgA (g/L)	0.85	0.84	0.7–4
IgM (g/L)	0.52	**0.35**	0.40–2.3
IgE (kU/L)	13	6.4	<100
Baseline Anti-TT (IU/mL)	1.3	3	>0.1
Anti-PnPS (U/mL, post-vaccination)	2 (non-protective)	NA	
Isohemagglutinin titers	Anti-A 1/32 Anti-B 1/64	Anti-A 1/64 Anti-B 1/32	≥1/8
CMV DNA PCR (copies/mL)	Negative	**1506**	Negative
EBV DNA PCR (copies/mL)	**810**	**1390**	Negative
EBV IgG	**Positive**	**Positive**	Positive after infection
Abnormal CDT Pattern	Not detected	Not detected	

ALT: Alanine Aminotransferase; AST: Aspartate Aminotransferase; Anti-TT: Anti-Tetanus Toxoid; CDT: Carbohydrate Deficient Transferrin; CMV: Cytomegalovirus; EBV: Epstein–Barr Virus; ELISA: Enzyme-Linked Immunosorbent Assay; Ig: Immunoglobulin; PCR: Polymerase Chain Reaction; PFA: Platelet Function Assay; PnPS: Pneumococcal Polysaccharide. Note: Bold values indicate results outside the reference range.

**Table 4:** Proposed descriptive and conceptual framework for the categorization of neurological involvement in XMEN disease.

Classification	Age at Onset	Neurological Features	Radiological Findings	Suggested Surveillance Focus
**TYPE I:** **Neuro-** **developmental** **(Childhood-onset)** (Synthesized from [4,6,8,9,11])	Pediatric	**Core (CDG-like) features** Speech and/or motor delay Global developmental delay ± ID Mild facial dysmorphism (subset) **Associated features (variable):** Learning difficulties Behavioral abnormalities Seizures (focal or generalized)	Cavum septum pellucidum Non-specific structural anomalies	Early developmental screening Speech and physical therapy EEG monitoring
**TYPE II:** **Neurodegenerative and Neuropsychiatric** **(Adult-onset/Progressive)** (Synthesized from [4,9,13,14] and the Current Study [P1])	Adult	**Neurodegenerative features** Progressive Ataxia ± Intention Tremor Spasticity and Extrapyramidal signs Cognitive decline **Neuropsychiatric features** Psychosis and Disinhibition Mood symptoms and Emotional lability	CNS atrophy Basal ganglia and Thalamic calcifications Fronto-subcortical white matter changes	Biennial brain MRI (volumetry ± GCA [Pasquier]) Serial neurological and cognitive assessments EEG as clinically indicated Psychiatric screening
**TYPE III:** **Secondary CNS and Peripheral** **(Infectious/** **Vascular/Immune)** (Synthesized from [4,8,9,10,12,22])	Variable	**Secondary CNS events:** Rapid cognitive decline (PML) Acute Encephalopathy Acute Visual Disturbances Stroke-like episodes and Hemiparesis **Peripheral neurological events:** Guillain-Barré Syndrome	Multifocal demyelinating lesions (PML) PRES (After chemotherapy) Vasculitic infarcts and Ischemic lesions Lymphoma involvement of the CNS	EBV monitoring ± targeted viral studies CSF analysis when indicated Urgent MRI/MRA/MRV for acute CNS events EMG/NCS for peripheral neurological symptoms Malignancy screening

CNS: Central Nervous System; CSF: Cerebrospinal Fluid; CDG: Congenital Disorders of Glycosylation; EBV: Epstein–Barr Virus; EEG: Electroencephalography; EMG/NCS: Electromyography/Nerve Conduction Studies; GCA: Global Cortical Atrophy (Pasquier scale); ID: Intellectual Disability; MRA/MRV: Magnetic Resonance Angiography/Venography; MRI: Magnetic Resonance Imaging; PET-CT: Positron Emission Tomography–Computed Tomography; PML: Progressive Multifocal Leukoencephalopathy; PRES: Posterior Reversible Encephalopathy Syndrome. Note: Clinical and radiological features listed represent cumulative findings reported in the literature for each proposed subtype and do not necessarily correspond to a single patient.

## Data Availability

The *MAGT1* variant data generated in the current study have been deposited in ClinVar under accession number SCV007293792. The remaining data that support the findings of this study are available from the corresponding author upon reasonable request.

## Supplementary Materials

The following supporting information can be downloaded at https://www.mdpi.com/article/10.3390/jcm15062395/s1. Figure S1: Expression patterns of COL4A1 and MAGT1 in human brain tissue; Table S1: In silico characteristics of the *COL4A1* variant identified in the index patient.

## Abbreviations

The following abbreviations are used in this manuscript:

ACMG	American College of Medical Genetics and Genomics
AIHA	Autoimmune Hemolytic Anemia
ALT	Alanine Aminotransferase
Anti-TT	Anti-Tetanus Toxoid
AST	Aspartate Aminotransferase
CADD	Combined Annotation Dependent Depletion
CDG	Congenital Disorders of Glycosylation
CDT	Carbohydrate-Deficient Transferrin
CM	Central Memory (T cells)
CMV	Cytomegalovirus
CNS	Central Nervous System
COL4A1	Collagen Type IV Alpha 1 chain
CSF	Cerebrospinal Fluid
DNT	Double-Negative T cells (TCRαβ^+^ CD4^−^ CD8^−^)
EBER	EBV-encoded small RNAs (in situ hybridization)
EBV	Epstein–Barr Virus
EEG	Electroencephalography
ELISA	Enzyme-Linked Immunosorbent Assay
EM	Effector Memory (T cells)
EMG/NCS	Electromyography/Nerve Conduction Studies
FLAIR	Fluid-Attenuated Inversion Recovery
GCA	Global Cortical Atrophy
gnomAD	Genome Aggregation Database
HSCT	Hematopoietic Stem Cell Transplantation
HSV	Herpes Simplex Virus
HUS/aHUS	Hemolytic Uremic Syndrome/Atypical HUS
ID	Intellectual Disability
ITP	Immune Thrombocytopenia
IVIG	Intravenous Immunoglobulin
MAGT1	Magnesium Transporter 1
MFI	Median Fluorescence Intensity
MRA/MRV	Magnetic Resonance Angiography/Venography
MRI	Magnetic Resonance Imaging
NK	Natural Killer (cells)
NKG2D	Natural Killer Group 2, member D
NLPHL	Nodular lymphocyte-predominant Hodgkin lymphoma
OST	Oligosaccharyltransferase complex
PBMC	Peripheral Blood Mononuclear Cells
PBS	Phosphate-Buffered Saline
PCR	Polymerase Chain Reaction
PET-CT	Positron Emission Tomography–Computed Tomography
PFA	Platelet Function Assay
PML	Progressive Multifocal Leukoencephalopathy
PnPS	Pneumococcal Polysaccharide
PRES	Posterior Reversible Encephalopathy Syndrome
R-CHOP	Rituximab, Cyclophosphamide, Doxorubicin, Vincristine, Prednisone
STT3B	Staurosporine-and-temperature-sensitive 3B
TCR	T-Cell Receptor
TEMRA	Terminally Differentiated Effector Memory re-expressing CD45RA
TMA	Thrombotic Microangiopathy
TUSC3	Tumor Suppressor Candidate 3
VUS	Variant of Uncertain Significance
vWF	von Willebrand factor
VZV	Varicella Zoster Virus
WES	Whole-Exome Sequencing
XMEN	X-linked immunodeficiency with magnesium defect, EBV infection, and neoplasia
