# Supplementary material for: Modifier-Sensitive Phenotypic Divergence in XMEN Disease (MAGT1 Deficiency): Neurodegenerative and Immuno-Hematologic Trajectories
Source: J Clin Med. 2026 Mar 21;15(6):2395. doi: 10.3390/jcm15062395 (PMC13028072; doi:10.3390/jcm15062395)
Supplement: Supplementary file 1 [file jcm-15-02395-s001.zip › jcm-4174424-supplementary.pdf]

Supplementary Table S1. In silico characteristics of the COL4A1 variant identified in the index patient.

Parameter	Result / Score	Interpretation
Variant	c.3662C>T (p.Pro1221Leu)	Missense variant
ClinVar	Uncertain Significance	No established pathogenic classification
Population frequency (gnomAD)	0.007% (Ultra-rare)	Extremely rare
Conservation (phyloP100)	2.108	Evolutionarily conserved residue
CADD Score	23.5	High predicted deleteriousness
Inheritance	De Novo	Absent in unaffected family members.
Expression Data	High in Arteries	High expression in vascular system

CADD: Combined Annotation Dependent Depletion; gnomAD: Genome Aggregation Database; NA: Not Applicable. Note: Predicted deleteriousness was evaluated using CADD (v1.6). Conservation scores represent phyloP 100-way vertebrate alignment.

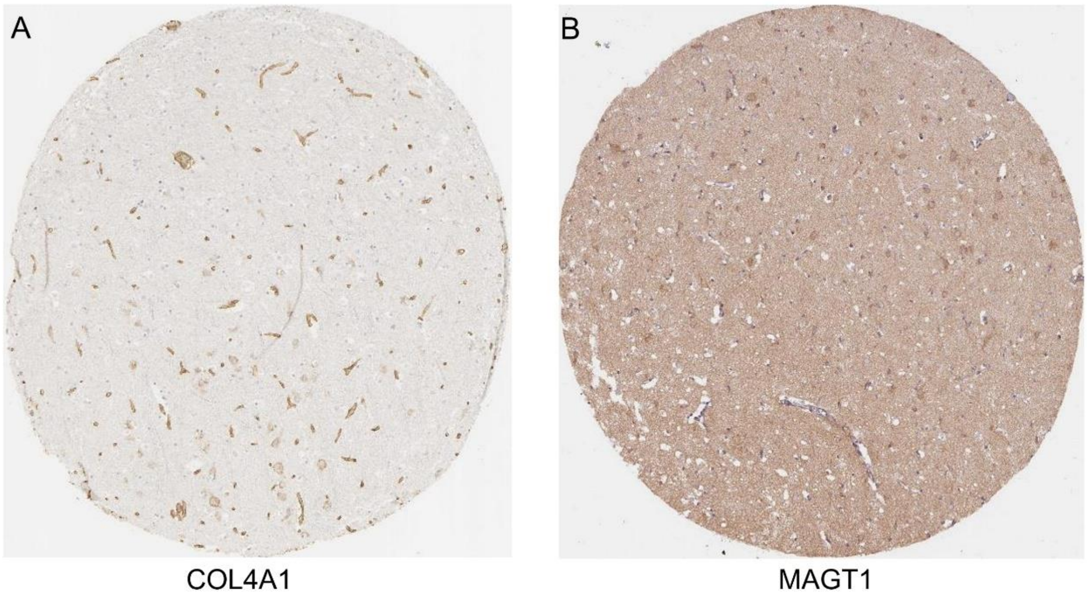

Supplementary Figure S1. Expression patterns of COL4A1 and MAGT1 in human brain tissue.

Representative immunohistochemistry images from the Human Protein Atlas illustrating (A) COL4A1 localization predominantly associated with vascular and basement membrane structures in cerebral microvessels, and (B) widespread MAGT1 expression in the human cerebral cortex. Images are provided for contextual support of the proposed glycosylation–matrix vulnerability axis and do not imply variant-specific or disease-specific pathology.

Images reproduced from the Human Protein Atlas (https://www.proteinatlas.org) under the Creative Commons Attribution-ShareAlike 4.0 International License (CC BY-SA 4.0).
